# Developing a Heart Transplantation Self-Management Support Mobile Health App in Taiwan: Qualitative Study

**DOI:** 10.2196/18999

**Published:** 2020-08-19

**Authors:** Yi-Wen Chen, Jeng Wei, Hwei-Ling Chen, Ching-Hui Cheng, I-Ching Hou

**Affiliations:** 1 Heart Center Cheng Hsin General Hospital Taipei Taiwan; 2 School of Nursing National Yang-Ming University Taipei Taiwan

**Keywords:** heart transplantation, mobile health app, self-management

## Abstract

**Background:**

Heart transplantation (HTx) is the most effective treatment for end-stage heart failure patients. After transplantation, patients face physiological, psychological, social, and other health care problems. Mobile health (mHealth) apps can change the delivery of conventional health care to ubiquitous care and improve health care quality. However, a dearth of mHealth apps exists for patients with HTx worldwide, including in Taiwan.

**Objective:**

The aim of this study was to investigate the information needed and to develop a preliminary framework for an mHealth app for post-HTx patients.

**Methods:**

A qualitative approach with individual in-depth interviews was conducted at a heart center in the regional hospital of northern Taiwan from June to November 2017. Patients that had undergone HTx and their health professionals were recruited for purposeful sampling. A semistructured interview guideline was used for individual interviews and transcribed. Thematic analysis was used for data analysis.

**Results:**

A total of 21 subjects, including 17 patients and 4 health professionals, were recruited for the study. The following five major themes were identified: reminding, querying, experience sharing, diet, and expert consulting. Minor themes included a desire to use the app with artificial intelligence and integration with professional management.

**Conclusions:**

An intelligent mHealth app that addresses the five main themes and integrates the processes of using a mobile app could facilitate HTx self-management for Taiwanese patients.

## Introduction

### Background

Heart transplantation (HTx) is a surgery for patients with heart failure who are not eligible for drug or other surgical treatments, involving mechanical assistance such as extracorporeal membrane oxygenation or a ventricular assist device, which is then replaced with a healthier heart [1,2]. Since Barnard performed the world’s first HTx in 1967 [3], according to the International Society for Heart and Lung Transplantation, there have been approximately 5000 HTx (adult and pediatric) performed every year worldwide [4]. The first HTx in Taiwan was performed in 1987 [5], and according to reports of the Taiwan Organ Registry and Sharing Center, there have been approximately 668 HTx performed to date in the country, with 1-, 3-, 5-, and 8-year allograft survival rates of 79%, 71%, 65%, and 56%, respectively [6].

After HTx, the recipients have to take immunosuppressants to decrease the activity of their immune system and prevent it from attacking the donated heart. The current standard immunosuppressive regimens include calcineurin inhibitors (eg, cyclosporine, tacrolimus, Prograf), antiproliferative agents (eg, azathioprine and mycophenolate mofetil), and steroids (eg, methylprednisolone, prednisolone) [7,8]. The main side effects of immunosuppressants include nephrotoxicity, hypertension, trembling, body hair growth, gum hypertrophy, liver toxicity, high blood sugar, high cholesterol, myelosuppression, leukopenia, thrombocytopenia, hypocytopenia, Cushing signs, weight gain, high blood pressure, high blood lipids, and gastrointestinal bleeding [9,10].

Previous studies showed that approximately 20%-30% of patients with HTx exhibit drug noncompliance [11] or forget to take their drugs [12]. Patient drug noncompliance increases with a longer time after HTx [13]. The main health problems caused by taking immunosuppressants after HTx are physical deterioration, fatigue, foot cramps, hair hyperplasia, moon face, poor vision, and acne, sequentially [14-17]. Anxiety and depression are the most common psychological problems in HTx patients [18]. The reasons for psychological stress include unclear prognosis of the disease, fear of death, fear of rejection, fear of complications, fear of increasing family trouble, and pressure to use drugs [19,20].

Currently, wireless networks, smartphones, and mobile health (mHealth) apps are becoming increasingly more popular. According to a systematic review literature, patients with cardiovascular disease, acquired immunodeficiency syndrome, diabetes, and organ transplants have positive perceptions after using mHealth services to improve their medication compliance [21]. In 2016, a mobile app for heart failure patients was developed, which included functions for self-assessment of heart failure symptoms, exercise recommendations, vital signs records, and statistical data. The graphics helped physicians make decisions and the research findings showed good results for patients to self-manage the disease, improve compliance with medications, and implementation of diet and exercise [22]. Online and smartphone-based apps for cardiac rehabilitation programs can augment secondary prevention strategies compared with standard cardiac rehabilitation. In particular, the app was shown to improve risk factors (eg, weight, blood pressure, and diet) and to reduce the health care burden of repeat cardiovascular disease events (eg, rate of rehospitalizations/emergency department visits) [23]. Patients with chronic diseases who used mobile phones (eg, medication reminder apps, text messaging) showed better medication adherence compared with usual care [24,25]. In a randomized 3-month study, patients with coronary heart disease who were found to have an app showed increased drug compliance, and patients with positive app acceptance and participation with the app showed positive results [26]. These studies demonstrated that mHealth apps can support patients with specific diseases for health self-management.

To our knowledge, there is no mHealth app available for supporting patients with HTx in Taiwan. Most hospitals use traditional self-management education and clinic consultation to support their patients. There are some disadvantages of this approach, including the fact that patients may not fully understand the interventions of self-management within the limited clinic visiting time and they often have a physical burden after surgery (eg, pain, fatigue). Thus, a tailored HTx mHealth app could offer continuous support to patients anytime and anywhere to ultimately improve their quality of life in the long term. To support patients with HTx, Cheng Hsin General Hospital (an HTx-specific hospital in Taiwan) and National Yang-Ming University have been working together to design an HTx mHealth app since 2017.

### Objectives

When developing an effective mHealth app to support health self-management, it is important to understand the expectations of end users in the early phase. The end users of the mHealth app designed in this study are patients with HTx. However, according to a previous study, HTx physicians and nurses were the major education providers for the self-management of HTx-related problems [17]. An mHealth app with management from health professionals may be beneficial to patients when developing a useful app. To our knowledge, there is a dearth of studies investigating the information needed for patients with HTx and their health professionals in the design of mHealth apps. To address these gaps, the objective of this study was to discover these needs to facilitate future development of an HTx mHealth app.

## Methods

### Study Design and Ethics

In this study, we used a qualitative approach involving individual interviews with HTx patients and their health professionals from June to November in 2017, with the goal of collecting and analyzing their information needs in an mHealth app for supporting HTx health self-management. The researchers (YW, IC, LF) are trained in qualitative academic research and have extensive experience in individual interviews. The clinical researchers from Cheng Hsin General Hospital included the cardiac surgeon and the superintendent (WJ), director of nursing (HL), and nurse supervisor (CH) who facilitated the process of this study.

This study complies with the Helsinki Declaration and was approved by the Institutional Review Board of the study site in Taipei before the study began [No. (596) 106-04].

### Study Site and Service Processes

The study site is The Heart Center of Cheng Hsin General Hospital, which specializes in the diagnosis, management, and treatment of cardiovascular diseases in northern Taiwan. Up to February 2020, a total of 523 patients successfully received HTx operations, 46 of whom have survived postsurgery for more than 20 years. This survival rate is the highest in Taiwan. The HTx health professional team (of the Cardiovascular Surgery Division) consists of two surgeons who are qualified instructors of HTx and three coordinators who are responsible for communication between health professionals and patients through the service process. The study site also employs nurse practitioners, who are nurses that assist with frontline surgery/medical therapy and direct care.

The service process for patients with HTx includes three phases: before hospital admission, during hospitalization, and at discharge. The first phase includes cases with acceptable preevaluation information (eg, medical records, test results). Consultation, laboratory tests, preoperative evaluations, surgery/medical therapy, intensive care, recuperation, and cardiac rehabilitation are included in the second phase. Follow-up services (eg, regular clinic appointments, telecommunication as needed) are provided in the third phase.

### Samples

**Figure 1:**
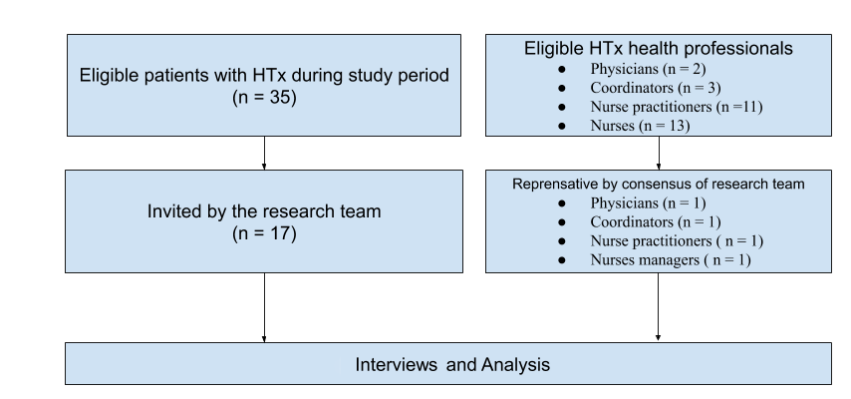
Study flowchart.

To fully understand the users’ information needs for the mHealth app, a purposeful sampling approach was used to recruit patients with HTx and their health professionals for this study. The inclusion criteria are described below and a flowchart of the sampling process is shown in Figure 1.

For patients with HTx, the inclusion criteria were: (1) HTx recipients who regularly visited the HTx clinic (Tuesday afternoons) during the study period (June to November 2017), (2) over 20 years old, (3) native Mandarin/Taiwanese speakers, and (4) mobile phone users. The exclusion criteria were: (1) mental illness and (2) slurred speech.The patients matched to the inclusion criteria were referred by the HTx coordinators to the researcher (YW) to confirm their willingness to participate and all agreed to have individual interviews. Before the formal interview, the consent form was filled out and the formal interview was held by the researcher for at least 30 minutes in the meeting room of the study site.

Four types of HTx health professionals (physicians, coordinators, nurse practitioners, and nurses) who were the primary health self-management education providers at the study site were recruited [17]. The inclusion criteria for each role were: (1) experience with HTx direct care, (2) experience with HTx administrative management (eg, director, head nurse, assistant nurse, leader), and (3) mobile phone users. The exclusion criteria were not current research team members (all authors). After the consensus meeting of the research team, the representative health professionals were invited and all agreed to have individual interviews by the researcher (YW) at their office. Every subject was given US $15-30 for compensation after the interview according to their interview time.

### Interview Guideline

One interview guideline was created to support the data collection. The first part was for patients, which included demographics (eg, education, marital status, occupation before and after HTx, time after HTx) and open questions for mHealth app self-management on HTx (eg, what difficulties have you faced after HTx and how do you think the mobile app could support you?). The second part was for HTx health professionals, with open questions about how mHealth apps could support patients with HTx. The interview guideline was reviewed by the nurse managers of the heart center at the study site (HL, CH), who confirmed the appropriateness before individual interviews. The details of the guideline are provided in Textbox 1.

**Details of the interview guideline for heart transplant (HTx) patients and professionals.**

**Interview guideline for patients with HTx**

- Name:
- Age:
- Education:
- Marital status:
- Occupation before HTx:
- Occupation after HTx:
- Time since HTx:
- Mobile smartphone experience: Yes/No
- Open questions:

1. Would you please tell me what difficulties you have faced after HTx (eg, medication side effects, nutrition intake, etc)?

2. We would like to develop a mobile phone app to support patients with HTx on self-management in the future. How do you think the mobile health app could support you (eg, useful functions)?

**Interview guideline for HTx professionals**

- Name:
- Age:
- Education:
- HTx working years:
- Open questions:

1. Would you please tell me what difficulties you have faced in managing patients with HTx (eg, medication compliance, communication problems, etc)?

2. We would like to develop a mobile phone health app to support patients with HTx on health self-management in the future. How do you think that mobile health apps could support them (eg, useful functions)?

### Data Collection

Before the interview, a brief introduction to the study and interviewer was provided by one of the researchers (YW). An electronic voice recorder was used with permission of the subjects. During individual interviews, the subjects were free to answer the questions and allowed to refuse when they felt uncomfortable. The researcher encouraged them to share their opinions without enforcement and interruption. Field notes were also taken by the interviewer to retrieve the key information from subjects. Before ending the interview, the subjects were asked whether they had additional comments to share and confirmed that there were no more data to be obtained from the subjects. After the interview, all voice records and nonverbal body language (eg, facial expressions, voice tone, and motions) were transcribed verbatim within 48 hours and returned (via email or post) to the subjects to confirm the semantic accuracy [27].

### Information Needs Analysis Framework

We adopted the methodology of Vaismoradi et al [28] for trustworthy thematic analysis to summarize our subjects’ information needs and to analyze the data from the individual interviews. The first step involved an overall reading of the text. All of the verbatim transcribed notes were read by researchers (YW, IC) with an open mind. Any meaningful text or researchers’ reflections were written down as the fundamental content for theme retrieving after the initial reading. The second step involved generating initial codes. According to the purpose of the study, the researchers generated the initial codes with empathy to the mHealth app needs of the subjects. The third step involved generating confirming codes. To clarify the contradictions and find hidden messages from the original verbatim transcribed text, the researchers read the text again. The initial codes were reconfirmed, adjusted, added, and any redundancies were eliminated. The fourth step involved generating the initial themes. The codes with similar meaning were categorized into the same themes. The fifth step involved generating the confirmed themes. After rechecking the verbatim transcribed text and reflecting on the journal and codes, the final themes and their names were confirmed by the research team. Finally, the report was produced. The Consolidated Criteria for Reporting Qualitative Research guidelines were used to produce this report. Through the process of analysis, peer debriefing, reflective journaling, and consensus of research teams, theme saturation was achieved. With the names of codes, themes were created according to familiarity for HTx patients, and these names also helped with communication for mHealth app development. Table 1 provides an example of the thematic analysis process followed in this study.

**Table 1:** Example analysis of information needs framework for the Reminder theme.

Transcribed text from interview	Codes
“When I am busy, the (mobile) phone (app) could remind me to take four FK (tacrolimus; FK506) in the morning and three (FK) in the afternoon.” -Patient B, 50-year-old man, college, business, CAD^a^ with ICMP^b^	First reminder for medication
“I used the alarm clock with my (mobile) phone to remind me (to take the medication) regularly and so did my wife. When I forgot (to take the medication), my wife would remind me (to take the medication).” -Patient L, 54-year-old man, senior high school, retirement, DCM^c^	Second reminder for medication
“When we decided to adjust the medication (the dose of immunosuppressant), we would send out messages (to patients) through Line message. The patients would know their medication was adjusted. (The hospital side would know the patient read the message).” - P1, 48-year-old man, college, heart transplant physician	Reminder of medication dose adjustment
“When patients return home after the heart transplant, the app would remind them about influenza if they have poor immunity. In the fall and winter, there would be a reminder warning about the window of the peak of influenza. Patients should pay attention to it.” - P3, 34-year-old woman, college, cardiac intensive care unit assistant head nurse	Reminder about influenza season

^a^CAD: coronary artery disease.

^b^ICMP: ischemic cardiomyopathy.

^c^DCM: dilated cardiomyopathy.

## Results

### Description of the Subjects

A total of 21 subjects, including 17 patients with HTx and 4 health professionals, were recruited from June to November 2017. Most of the patients were male (13/17, 77%), 51 to 60 years old (7/17, 41%), with a college education (8/17, 47%), married (11/17, 65%), had a job before HTx (15/17, 88%) and after HTx (8/17, 47%), were diagnosed with dilated cardiomyopathy (DCM) (14/17, 82%), and received HTx more than 2 years previously (10/17, 59%). The detailed demographic data for each patient are shown in Table 2.

**Table 2:** Demographic data of heart transplant (HTx) patients.

Patient ID	Sex	Age (years)	Education	Marital status	Pre/post HTx occupation	Pre HTx diagnosis	Time since HTx
A	M	53	Master	Married	Business	CAD^a^ with AMI^b^	1-2 years
B	M	50	College	Married	Business	CAD with ICMP^c^	>2 years
C	M	39	College	Single	Service industry	DCM^d^	>2 years
D	F	43	College	Single	Service industry	DCM	>2 years
E	M	52	Junior high school	Single	Industry	DCM	>2 years
F	M	54	Junior high school	Married	Industry/Retired	DCM	<6 months
G	F	51	Junior college	Married	Service industry	DCM	>2 years
H	F	62	Elementary school	Married	Industry/Housewife	DCM	<6 months
I	M	55	College	Divorced	Service industry	DCM	>2 years
J	M	46	College	Divorced	Service industry/Retired	DCM	>2 years
K	F	50	Senior high school	Married	Housewife	DCM	>2 years
L	M	54	Senior high school	Married	Industry/Retired	DCM	>2 years
M	M	27	College	Single	Unemployed	HOCM^e^	>2 years
N	M	32	College	Married	Service industry	DCM	1-1.5 years
O	M	55	Senior high school	Married	Service industry	DCM	1-1.5 years
P	M	48	College	Married	Business	DCM	1-1.5 years
Q	M	42	Junior high school	Married	Industry/Unemployed	DCM	1-1.5 years

^a^CAD: coronary artery disease.

^b^AMI: acute myocardial infarction.

^c^ICMP: ischemic cardiomyopathy.

^d^DCM: dilated cardiomyopathy.

^e^HOCM: hypertrophic cardiomyopathy.

The health professionals included one physician and three nurses who all had college degrees. The physician had more than 10 years of experience with HTx and is the director of the Taiwan Heart Transplant Association. The first nurse is the coordinator (ie, case manager) who had 11 years of experience with HTx direct care and currently communicates with patients, families, and hospitals. She is also responsible for outpatient clinic services, patient medication education, and holds support group activities for patients with HTx. The second nurse is the assistant head nurse in the cardiac intensive care unit who had 9 years of experience in intensive care for post HTx patients. The third nurse is the cardiac surgery nurse practitioner who had 10 years of experience and is responsible for the HTx care plan and evaluation. Their demographic data are shown in Table 3.

**Table 3:** Demographic characteristics of cardiology professionals.

ID	Sex	Age (years)	Education	Job title	Years of heart transplant experience
P1	M	48	College	Heart transplant physician	10
P2	F	44	College	Heart transplant coordinator	11
P3	F	34	College	Cardiac intensive care unit assistant head nurse	9
P4	F	34	College	Cardiac surgery nurse practitioner	10

### Main Themes

After the thematic analysis, a total of five major themes and 14 codes were identified (Textbox 2).

#### Theme 1: Reminder

Nearly every subject mentioned that the app would be helpful for reminding them to take their medication regularly. The alarm on a mobile phone is the most commonly used tool for preventing medication noncompliance. However, if they still forget to take the medication after turning off the alarm, the app with a medication reminder would be helpful for increasing their medicine compliance.

**Themes and codes as the framework for a heart transplant self-management app.**

**Theme 1: Reminder**

- 1-1. First reminder for regular medication administration
- 1-2. Second reminder for regular medication administration
- 1-3. Reminder when medication dose is adjusted
- 1-4. Reminder to return to the clinic for examination
- 1-5. Reminder of influenza season

**Theme 2: Query**

- 2-1. Laboratory results
- 2-2. Plasma drug concentration
- 2-3. Record of heart rate and blood pressure

**Theme 3: Experience Sharing**

- 3-1. Asynchronous experience sharing
- 3-2. Synchronous experience sharing

**Theme 4: Diet**

- 4-1. Diet guideline
- 4-2. Recipes

**Theme 5: Expert Consulting**

- 5-1. Artificial intelligence consulting
- 5-2. Remote professional consulting

##### Code 1-1: First Reminder for Regular Medication Administration

It is good to have a reminder (in the app), the mobile phone can help patients to take medicine regularly and decrease the risk of transplant rejection.

P2, 44-year-old woman; heart transplant coordinator

The (mobile) phone (app) could remind patients to take four FK (tacrolimus; FK506) in the morning and three (FK) in the afternoon.

Patient B, 50-year-old man, college, business, coronary artery disease (CAD) with ischemic cardiomyopathy

For those elderly (patients), sometimes they forget (to take medication). If there is an app to remind you to take the dose of medicine in the mornings, afternoons, and evenings, it would be really helpful.

Patient I, 55-year-old man, college, service industry, DCM

In addition to the reminder to take medication, the patients with HTx also mentioned that they need the app to provide drug information since there are so many different kinds of medications to take after HTx. They can better follow the instructions of taking the medication if they have more knowledge of them.

The app should not only remind the dose of medication for each person to take but also provide the image, action, and side effects of the drug. Then, I would feel more clarity.

Patient C, 39-year-old man, college, service industry, DCM

There should be the label and image of the drug on the screen of the mobile phone to remind me to take the medicine. That would prevent me from taking the wrong medication.

Patient Q, 42-year-old man, junior high school, industry before HTx and unemployed after HTx, DCM

##### Code 1-2: Second Reminder for Regular Medication Administration

Some patients with HTx also mentioned that they depend on their family to remind them to take medication. The secondary reminder would provide them with more confidence to prevent the undesired neglect to take medication.

I use the alarm clock with my (mobile) phone to remind me (to take the medication) regularly. My wife also does the same thing. When I forgot (to take the medication) at the time, my wife also reminds me (to take the medication).

Patient L, 54-year-old man, senior high school, retired, DCM

I am afraid of forgetting to take so many medications and I need my husband to remind me. If the phone app rings (referring to having the reminder from the app), then I will take the medication. It is convenient for my husband’s phone app to ring to make sure I take the medication.

Patient H, 62-year-old man, elementary school, industry before HTx and housewife after HTx, DCM

##### Code 1-3: Reminder When Medication Dose is Adjusted

In addition to the reminder for needing to take medication regularly, having information on medication dose adjustment according to their plasma immunosuppressants concentration is also important for patients with HTx. The medication dose adjustment reminder is currently provided by the HTx coordinator via telephone when their laboratory report is received. After HTx patients return to the outpatient clinic and have their blood drawn, the physician then adjusts their immunosuppressants dose according to their plasma drug concentration. However, there can be problems such as the patients being out of reach (eg, too busy to answer the phone) or forgetting the verbal order, and thus taking the wrong dose. In these situations, the subjects would like the app to remind them when medication dose adjustment is needed and to improve communication between patients and health professionals.

I think it (the reminder of medication dose adjustment) is necessary, especially at the beginning of receiving HTx. It would be convenient to receive the drug adjustment message from the app because sometimes (I) would forget.

Patient G, 51-year-old man, junior college, service industry, DCM

When we decide to adjust medication (the dose of immunosuppressant), we could send out a message (to patients) through the Line message. The patient would know how their medication should be adjusted. (The hospital side would know the patient read the message.)

P1, 48-year-old man, college, HTx physician

##### Code 1-4: Reminder to Return to the Clinic for Examination

Some subjects also mentioned that the app should provide the reminder of the outpatient clinic and examination appointments to support the patients and health professionals.

I do not know if it’s because of retirement, but my memory seems to be getting worse. So, if the phone (app) can remind me to return (to the clinic), my wife and I would have less trouble.

Patient J, 46-year-old man, college, service industry before HTx and retired after HTx, DCM

For the reminders on clinic appointments, if the app could remind them (HTx patients) to return (clinic appointment) after making the reminder setting, the following reminders are automatic (without setting manually), which could save lots of time for the (HTx) coordinator. They only (need to) find out which patients had no response (those who do not return to the clinic for follow up) and focus on reminding them.

P1, 48-year-old man, college, HTx physician

##### Code 1-5: Reminder of Influenza Season

The subjects also mentioned that the app could send a warning or voice reminder during influenza season to support HTx self-care taking into account their lower immune system and provide self-protection procedures.

I am worried about getting an infection. So, (the app) could remind me to wear a mask during influenza season.

Patient O, 55-year-old man, senior high school, service industry before HTx and unemployed after HTx, DCM

It is easy to catch a cold when the seasons alternate. The app can remind me there is an influenza outbreak.

Patient P, 48-year-old man, college, business, DCM

I think when patients return home, there should be a reminder about the influenza season when their immune systems are weak. In the fall and winter seasons, the app should have a pop-up reminder that it’s the influenza season and to be careful.

P3, 34-year-old woman, cardiac intensive care unit assistant head nurse

#### Theme 2: Query

Over half of the subjects indicated that they would like the app to provide query functions for laboratory results (eg, liver function index, renal function index, hemoglobin, hematocrit, blood sugar), plasma drug concentrations, heart rate records, and blood pressure. Such functions are convenient for patients with HTx to take care of themselves and they also could show their laboratory data to their physician when they return for a clinic visit. Some patients with HTx also felt that such functions would be more helpful than the oral report from health professionals.

##### Code 2-1: Laboratory Results

I hope I could query about my (laboratory) report of blood drawing in my phone (app) such as hemoglobin and liver function index. (Referring to his mobile phone with his finger and nodding.)

Patient M, 27-year-old man, college, unemployed, hypertrophic cardiomyopathy (HOCM)

If queries are provided in the app, that would be better than the (HTx coordinator) and ribbit (nagging) you. You can remind yourself what the query of the last examination report was. (The subject refers to the coordinator with their finger with a smile on their face.)

Patient B, 50-year-old man, college, business, CAD

##### Code 2-2: Plasma Drug Concentration

Many subjects queried about the report of their plasma drug concentrations and indicated that showing the trend in the app was very important to them. Such functions could show them the importance of taking immunosuppressants regularly and prevent rejection.

I could see data of my drug plasma concentration (from the app). It is very important for me to know the importance of taking medication.

Patient M, 27-year-old man, college, unemployed, HOCM

To see the figure curves of drug plasma concentration would be very convenient for the patient to return to the clinic and show their physicians.

P4, 34-year-old man, college, cardiac surgery nurse practitioner

##### Code 2-3: Record of Heart Rate and Blood Pressure

Some of the subjects mentioned that the app should provide records of heart rate and blood pressure as an important index of heart function, which would help them to better control their body.

If the app could integrate with other devices then I can check my blood pressure and heart rate, which would be better. (The subject refers to the data check in the monitor in the hospital which could be integrated in the app)

Patient C, 39 year-old-man, college, service industry, DCM

I think there should be basic blood pressure and heart rate monitoring and then I could see the results. It would be very convenient.

Patient D, 43-year-old man, college, service industry, DCM

#### Theme 3: Experience Sharing

About half of the subjects mentioned that would like to share their experience and information with other HTx patients asynchronously (eg, via a blog) or synchronously (eg, in an online chatting room). For new patients with HTx, asynchronous experience sharing would help them learn more about how to take care of themselves from a more senior patient with HTx.

##### Code 3-1: Asynchronous Experience Sharing

I told my psychologist about the panic attack (the subject felt chest tightness and nervous) at midnight. He encouraged me to write down the severity of anxiety before the heart biopsy. Now, I am creating a blog and hope to record the process through texts. The blog could share important messages with patients and families. Such peer groups are very important.

Patient A, 53-year-old man, master degree, business, CAD

It is good (referring to the experience sharing post HTx). We can learn from other people how to protect their heart. My husband and I can pay attention to these experiences.

Patient H, 62-year-old woman, elementary school, housewife, DCM

##### Code 3-2: Synchronous Experience Sharing

Synchronous experience sharing could support patients mentally (eg, to decrease the anxiety on the uncertainty of a rejection response) and prevent social isolation.

We understand that new people (referring to new patients with HTx) on the medication and other problems would be more anxious and worry about their condition. To have such (experience sharing), would be helpful.

Patient G, 51-year-old man, junior college, service industry, DCM

You may have less contact with your friends. I participated in many activities before, but after HTx, there may be foods I cannot eat. Maybe friends go to climb a (mountain) or have high-intensity activities and we may not be able to participate. Then, we become far away from friends. If the app can provide a chatting room, it allows the patients to have a place where they can decompress.

Patient K, 50-year-old woman, senior high school, housewife, DCM

#### Theme 4: Diet

Some of subjects mentioned they would like to know about the diet guidelines after HTx. They were told that they cannot eat certain foods without cooking them first such as raw fish and salads, which they liked to eat before HTx. They felt the limitations of food choices in their daily life. They would like to know what foods they can eat and to tailor the food recommendations according to their physiques.

##### Code 4-1: Diet Guidelines

I think there should be customized functions in the app which provides recommendations on the nutrition according to your physique.

Patient A, 53-year-old man, master degree, business, CAD

There should be a notice (in the app) on which raw foods to avoid and if grapefruits would affect the metabolizing of the medication.

Patient E, 52-year-old man, junior high school, industry, DCM

We, the earlier HTx patients, were told by the health professionals that we could not take Chinese herbs if we needed to inactivate our immune system (referring to preventing an auto rejection response). Some Chinese herbs would activate the immune system of the body like wheat grass juice. Such knowledge should be provided earlier for our specific body condition and prevent us from eating those foods.

Patient G, 51-year-old man, junior college, service industry, DCM

I hope there would be a list of foods that we can eat and I can see the detailed information when I click on it.

Patient N, 32-year-old man, college, service industry before HTx and unemployed after HTx, DCM

##### Code 4-2: Recipes

Some of the subjects had the problem of becoming overweight after HTx from taking steroids. They need the app to provide recipes, calculate food calories, and determine the daily intake of calories.

I think you can provide food recipes. Recently, I read the recipes for patients after HTx. The app could provide such recipes like a low-fat diet or low carbohydrate diet of your choice. You could provide recipes for 1 week and they could try them out. You can retrieve some recipes or food photos from the internet which I think would be good.

Patient K, 50-year-old woman, senior high school, housewife, DCM

The app could provide recipes or videos of cooking. Then, the patients would know what to eat when they come home.

P3, 34-year-old woman, cardiac intensive care unit assistant head nurse

#### Theme 5: Expert Consulting

The subjects indicated that the app could provide artificial intelligence consulting. The patients could upload a text or photo to the app for consulting. The app could then automatically answer the patients with efficiency.

##### Code 5-1: Artificial Intelligence Consulting

Like a bank app, when typing in simple questions about specific items, the app could automatically answer the questions.

Patient Q, 42-year-old man, junior high school, industry before HTx and unemployed after HTx, DCM

The app could let the patients ask questions with a short text message. For those simple questions, the app could automatically respond to patients.

P4, 34-year-old woman, college, cardiac surgery nurse practitioner

##### Code 5-2: Remote Professional Consulting

For urgent situations, the subjects mentioned that they could remotely consult the health professionals through the telephone using photos, which can then be sent to the physician or coordinators for assessment and problem solving. Such functions would help patients who live in more remote areas to save on travel time.

If you have a problem, you can ask questions (with the app). For example, at midnight, when there is an urgent situation, some health professionals (in the app) can help.

Patient D, 43-year-old man, college, service industry, DCM

I think the phone number of HTx health professionals could be shown in the app for contact.

Patient N, 32-year-old man, college, service industry before HTx and unemployed after HTx, DCM

When patients come home, there could be a health problem like limb edema from their heart function getting worse. Patients who live in the middle or south of Taiwan could send photos to ask the physician or coordinator. That would save on their travel time and solve the problem immediately.

P3, 34-year-old woman, cardiac intensive care unit assistant head nurse

## Discussion

### Principal Findings and Comparison to Previous Studies

According to the results, the information needed for patients with HTx on mHealth apps included five main themes: reminder, query, experience sharing, and diet and expert consulting. The results were mainly consistent with a previous study showing that patients with HTx need self-management education such as about regular medication taken, regular exercise, diet control, infection/rejection signs observation, and regular clinic visiting, which could support patients with HTx on health self-management [29].

Most subjects indicated needing the app to support them on reminders for medication taken (code 1-1, code 1-2, code 1-3), clinic/examination appointments (code 1-4), and influenza season (code 1-5). With regard to the reminder for medication taken, this is focused on immunosuppressants management to prevent rejection of the grafted heart [7,8], supporting the same importance and difficulty in medication compliance as highlighted in previous studies [21]. To our knowledge, there are existing mHealth apps for supporting taking medication regularly [30] but these were not adopted by our subjects. Instead, they used the original alarm in the mobile phone but sometimes also forgot to take the medication. In such situations, some of their family members would remind them. The design of an mHealth app that could support secondary medication reminders from family or health professionals might be useful for such unexpected noncompliant medication situations.

To resolve the ineffective communication process between discharged patients and their health professionals (eg, informing medication adjustment, clinic appointments), our health professionals suggested that the app could support them in knowing that their messages had been received by the patients. Currently, such functions are provided by instant message software in Taiwan. Therefore, the newly developed app should consider involving the same function that may facilitate its adoption.

In addition, a reminder for influenza season (code 1-5) was indicated as a requirement of the app by both the patients and health professionals. The growing availability of big data in health care and public health opens up possibilities for infectious disease control in local settings. The detection and prediction (nowcasting) of influenza epidemics are now becoming possible [31].

With regard to the query of their laboratory report (code 2-1), plasma drug concentration (code 2-2), and their own health records (code 2-3), these needs are consistent with those of patients with other diseases [22,32]. Currently, most patients only know about their report when they come to the clinic and are told by the health professionals through an electronic health record query. Query from the app and the ability to read their report anytime may support patients in achieving self-control of their health behaviors rather than being passively monitored by health professionals.

With regard to the theme of experience sharing, our subjects had difficulties in facing activities with people owing to suffering from physical problems after HTx (eg, fatigue, Cushing syndrome) [14-17]. Therefore, the app may support patients in providing social connections with other patients to mentally support each other and decrease feelings of social distance anytime and anywhere (code 3-1, code 3-2). To our knowledge, social networks are popular (eg, Facebook, Instagram, Line) and most patients use them to exchange information. However, the individual privacy and accuracy of the content is a concern, as mentioned by some subjects: “There should be someone to manage the accuracy of the data” (Patient B, 50-year-old man, college, business, CAD); “If there is an online community, you need to manage the personal data, but who has that responsibility?” (Patient I, 55-year-old man, college, service industry, DCM).

With regard to the theme of diet, this is a common need expressed by patients who experience severe illnesses and recovery. To date, there have been many new technologies (eg, web-based tools, smartphone apps) developed to support diet self-management [33]. However, the diet guideline (code 4-1) specific to the needs of HTx patients (eg, avoid eating raw foods that may weaken their poor immune system) should be considered. Specifically, female subjects indicated a need for more advanced diet and caloric calculation functions in the app given concern for body weight control problems from taking steroid medications. Providing specific and customized recipes (code 4-2) in the app for patients with HTx may support them in consuming appropriate foods and obtaining good nutrition.

With regard to the theme of expert consulting, continuous support from health professionals was highlighted as very important to patients with HTx (code 5-1, code 5-2). A previous study showed that telemedicine apps can support communication about oral conditions among clinicians and patients [34]. Considering the busy work and limited human power of health professionals, the subjects referred to their experience of using artificial intelligence (eg, chat robot) and recommended the research team to design such functions in the app.

When patients with HTx have an emergency physical situation, an automatic quick response from the app when they ask questions can first help to screen the severity of their medical needs and save travel time on returning to the hospital. An app with artificial intelligence could increase the convenience. However, the validity and reliability of artificial intelligence should be evaluated carefully when adopted in the app to prevent risks. In addition, the app could facilitate communication between health professionals and patients. With the reports from the hospital’s side (eg, blood report, echo image, heart biopsy) and patient’s side (eg, body temperature, heart rate, blood pressure), both sides could easily communicate with each other through the app (eg, medical advice when there is an abnormal report). Such communication depends on integration into the hospital management in the future. These two minor findings would facilitate end user intent to use the app.

In summary, five main themes were identified by the research team. The information based on these main themes and their codes were then used to form the framework (Textbox 2) for development of an HTx mHealth self-management support app. Most themes were consistent with previous studies, but some new advanced codes specific to HTx management were uncovered (code 1-2, second reminder for regular medication administration; code 1-5, reminder of influenza season; code 5-1, artificial intelligence consulting). We believe that individual interviews with patients and health professionals is a strong method for identifying mHealth technology information needs for Taiwanese patients with HTx.

### Limitations

The first limitation of this study is that it was conducted in urban areas with more medical resources than are typically available in the more rural areas of the country, which might have resulted in geographical bias. We did not provide the simulation app, which might have resulted in different feedback. The second limitation of this study is the purposeful sampling approach, which prevented recruitment of more subjects and we did not consider the sex ratio among HTx patients, which might result in different information needed in the mHealth app. The third limitation is that we did not include the perspective from managers and information communication technology specialists at the study site, who may have insight into the policy (eg, human power to support an mHealth app service) and technology feasibility (eg, artificial intelligence consulting) on developing a patient-centered app to support HTx self-management.

### Summary and Conclusions

Our team used individual in-depth interviews to retrieve the information needed for use in an mHealth app from patients with HTx in Taiwan. A total of five main app needs were retrieved efficiently to facilitate developing an HTx self-management app. The next steps include building a real app and validating the self-management outcomes (eg, technology acceptance, medication compliance, rejection response, emergency visiting) from Taiwanese patients with HTx.

## Abbreviations

CAD: coronary artery disease
DCM: dilated cardiomyopathy
HOCM: hypertrophic cardiomyopathy
HTx: heart transplantation
mHealth: mobile health
